# Multimodal human action recognition and personalized sports health promotion: a deep learning framework integrating wearable sensor fusion

**DOI:** 10.3389/fnbot.2026.1785114

**Published:** 2026-04-10

**Authors:** Ying Xi, Taibin Huang, Zhiyu Yang

**Affiliations:** School of Physical, Xinyu University, Xinyu, Jiangxi, China

**Keywords:** deep learning, multimodal human action recognition, personalized intervention, sports health analytics, transformer-GCN, wearable sensor fusion

## Abstract

**Introduction:**

In real-world sports scenarios, Human Action Recognition (HAR) is often hindered by data complexity, limited dynamic adaptability, and fragmented integration of physiological and kinematic information. To address these challenges, this study proposes a multimodal HAR framework for personalized sports health promotion by integrating wearable sensor streams with deep learning architectures.

**Methods:**

The proposed system employs a robust sensing layer to capture 12-dimensional multimodal data and synchronize physiological indicators with behavioral signals in real time. A novel Transformer-GCN hybrid model was developed to extract complex spatiotemporal dependencies for accurate action recognition and dynamic state analysis. In addition, a reinforcement learning module was incorporated to generate adaptive exercise prescriptions based on user progress. The framework was deployed through a responsive interface for real-time intervention and evaluated in a 12-week randomized controlled trial.

**Results:**

The results demonstrated that the proposed framework achieved effective multimodal fusion and reliable action recognition in sports scenarios. After the 12-week intervention, participants in the intervention group showed a 20.1% increase in cardiorespiratory fitness (*VO*_2_ max), a 99.3% improvement in muscular endurance, and a sports injury rate maintained below 15%. These findings indicate that the framework can support accurate motion analysis and safe, personalized intervention.

**Discussion:**

The proposed multimodal fusion architecture effectively bridges the gap between action recognition and personalized sports health intervention. By combining wearable sensing, hybrid deep learning, and reinforcement learning, the framework provides a practical solution for AI-driven motion analysis and adaptive health promotion in land sports scenarios.

## 1 Introduction

Today, as the demands for population health management become increasingly sophisticated, sports health monitoring and analysis technology is undergoing profound changes from qualitative assessment to quantitative intervention. Traditional health management relies mostly on periodic physical examinations and subjective experience assessments, which are difficult to capture the details of the human body’s continuous physiological fluctuations and action patterns in real sports scenarios. With the popularization of wearable sensing technology, it has become possible to obtain high-temporal resolution human kinematics and physiological data, which provides a new perspective for understanding health risks and adaptation laws during exercise. However, how to transform these multi-dimensional data into effective health management strategies, that is, to achieve a closed loop from “monitoring” to “analysis” to “intervention,” is still a core challenge facing the current field of sports science.

The rising prevalence of chronic diseases and the global demand for precision health management have exposed the limitations of traditional “one-size-fits-all” exercise interventions. Despite the proliferation of digital health platforms, most still struggle to bridge the gap between static data collection and dynamic, personalized adjustments. Human Activity Recognition (HAR), a key technology for understanding human behavior, aims to automatically identify and classify human activity patterns through sensor data. Traditional HAR methods heavily rely on camera-based visual recognition, which is susceptible to changes in lighting, occlusion, and viewing angle limitations, and suffers from poor portability and privacy breaches. In contrast, wearable sensor-based HAR, especially methods that fuse multimodal sensor data, offers more discreet, continuous, and environmentally unconstrained monitoring capabilities. The fusion of multimodal wearable sensors and deep learning provides a solution for capturing complex physiological and movement patterns in real time. The rise of deep learning technology has further propelled the development of HAR. Deep learning possesses powerful end-to-end feature learning capabilities, enabling it to automatically extract high-level, discriminative spatiotemporal features from raw multimodal sensor data, thereby significantly improving the accuracy and robustness of complex action recognition. However, simply collecting data is insufficient; the real challenge lies in effectively fusing high-dimensional sensor data streams to achieve robust HAR in a scalable architecture and provide users with immediate and actionable biofeedback (Kumar et al., 2024; Saleem et al., 2023).

Deploying such a system in real-world environments presents significant technical hurdles, particularly regarding the synchronization of heterogeneous data streams and the latency of deep learning inference. To address these bottlenecks, we engineered a multimodal fusion framework deployed via a high-performance Web architecture. Our preprocessing pipeline tackles signal noise and misalignment through wavelet thresholding and LSTM-based imputation. The core innovation, however, is the hybrid analysis layer: we integrate a Transformer–GCN hybrid model to capture the spatiotemporal dependencies of human movement, embedded within a Federated Learning protocol to ensure user data privacy. Furthermore, to move beyond passive monitoring, we implemented a Proximal Policy Optimization (PPO) reinforcement learning module that dynamically adjusts exercise prescriptions based on the recognized fatigue states and performance metrics.

Despite significant technological advances, a persistent gap remains between accurate activity recognition and the delivery of actionable, personalized interventions (Afsar et al., 2023; Yang et al., 2024). While researchers have explored personalized optimization models—such as Chen et al. (2024) utilizing power load parameters and Lv et al. (2025) employing Long Short-Term Memory (LSTM) networks—few systems have achieved true real-time dynamic adaptation. Current predictive models (Alghamdi, 2023; Li et al., 2021) and fitness evaluation algorithms (Wu and Ouyang, 2025) frequently operate offline or lack the integration of continuous biofeedback loops.

Recent investigations have begun to explore the integration of multimodal sensor fusion and advanced artificial intelligence to bridge this gap (Mahato et al., 2024; Wu et al., 2025). Building upon Li’s (2025) vision of utilizing deep learning to promote health through physical training and the personalized sustainability focus discussed by Panahi (2025), this study proposes a unified framework. By integrating Transformer-GCN architectures with multimodal sensor fusion (Chung et al., 2019; Huynh-The et al., 2020), we aim to deliver a system that not only recognizes complex actions with high precision (Chen and Fan, 2025) but also generates real-time, personalized exercise prescriptions designed to minimize injury risk and maximize training efficiency (Wang et al., 2024).

This study makes three primary contributions to the field of intelligent sports analytics. First, we critically evaluate existing gaps in multimodal data fusion for health applications, identifying specific deficiencies in real-time adaptability. Second, we propose a novel end-to-end framework that seamlessly integrates sensor data acquisition, Transformer–GCN hybrid model-based HAR, and reinforcement learning decision-making, ensuring both high accuracy and system scalability. Third, the system’s efficacy is validated through a 12-week Randomized Controlled Trial (RCT), where we rigorously assess not just system metrics (latency, recognition accuracy) but, crucially, the tangible physiological improvements (VO_2_ max, muscular endurance) and injury prevention capabilities in active users.

## 2 Related work

The advancement of sports health promotion has increasingly relied on the convergence of wearable sensing technologies and artificial intelligence. This section reviews the evolution from basic sensor monitoring to complex, deep learning-driven Human Action Recognition (HAR) systems, highlighting gaps that the proposed framework aims to address. The proposed framework is also supported by previous studies on multimodal sensing, intelligent action recognition, physiological monitoring, and adaptive health intervention in sports and rehabilitation settings (Bianchi et al., 2019; Chao et al., 2024; Qiu et al., 2025; Tileubay et al., 2024; Yoon et al., 2021; Rinaldi et al., 2026; Suglia et al., 2026).

### 2.1 Evolution of wearable sensors in health monitoring

The foundation of modern sports analytics lies in the capability to capture high-fidelity physiological and kinematic data. Early interventions primarily utilized basic power load parameters to categorize cardiovascular risks (Chen et al., 2024). However, recent material science innovations have significantly expanded sensor capabilities. For instance, silk-based flexible sensors (Du et al., 2024) and piezoelectric composites made from PVDF/BaTiO_3_ (Su et al., 2024) have enabled non-invasive, high-precision detection of human movement. Similarly, innovations in fiber optic sensors (Zheng et al., 2024) and laser-induced graphene (Raza et al., 2022) have improved the sensitivity and durability required for rigorous athletic environments. These hardware advancements facilitate the collection of diverse data types, from heart rate variability in tennis training (Fan and Ji, 2024) to comprehensive physiological signals in smart clothing frameworks (Ahsan et al., 2022). As noted by Drew et al. (2023) and Bakker (2023), the integration of these sensing technologies into holistic health systems is critical for protecting athlete health and optimizing performance.

### 2.2 Multimodal data fusion and IoT integration

Single-sensor mode often struggles to capture the complexity of human motion in real-world scenarios. The workflow of a single modality faces several inherent limitations: First, it lacks the ability to finely distinguish movements; in complex environments or where motion artifacts are present, the signal-to-noise ratio of a single-source signal drops sharply. Second, it cannot fully reflect the physiological effects of motion. Consequently, research has shifted towards multimodal fusion. Mahato et al. (2024) demonstrated the efficacy of hybrid sensors in comprehensive monitoring, while Li et al. (2021) and Yang et al. (2024) emphasized the role of the Internet of Things (IoT) in transmitting this fused data for real-time analysis. The challenge remains in processing this heterogeneous data efficiently. Approaches like King et al.'s (2017) data fusion techniques and Chung et al.'s (2019) multimodal acquisition frameworks have laid the groundwork. Furthermore, contextual integration—such as combining real-time location systems with body sensors (Phatak et al., 2021) or merging sensor data with Virtual Reality (VR) environments (Li et al., 2024)—has shown promise in enhancing the ecological validity of training simulations.

### 2.3 Deep learning for human action recognition (HAR)

The application of Deep Learning (DL) has revolutionized the interpretation of sensor data, enabling robust HAR. Convolutional Neural Networks (CNNs) and Long Short-Term Memory (LSTM) networks are widely adopted for their ability to handle feature engineering and temporal sequence modeling. For example, Zhang et al. (2022) and Ascioglu and Senol (2020) reviewed how DL techniques significantly outperform traditional machine learning in activity recognition accuracy. Specific architectures have been developed for varied tasks: Chen and Fan (2025) optimized CNNs for exercise posture recognition, while Wang et al. (2023) combined CNN-LSTM with self-attention mechanisms to capture long-range dependencies in physiological signals. Additionally, newer paradigms like Afsar et al.’s (2023) use of DL in exergaming and Omarov et al.’s (2023) deep neural networks for physical training analysis highlight the trend towards end-to-end learning architectures (Ates et al., 2022).

## 3 Materials and methods

### 3.1 System architecture

**Figure 1:**
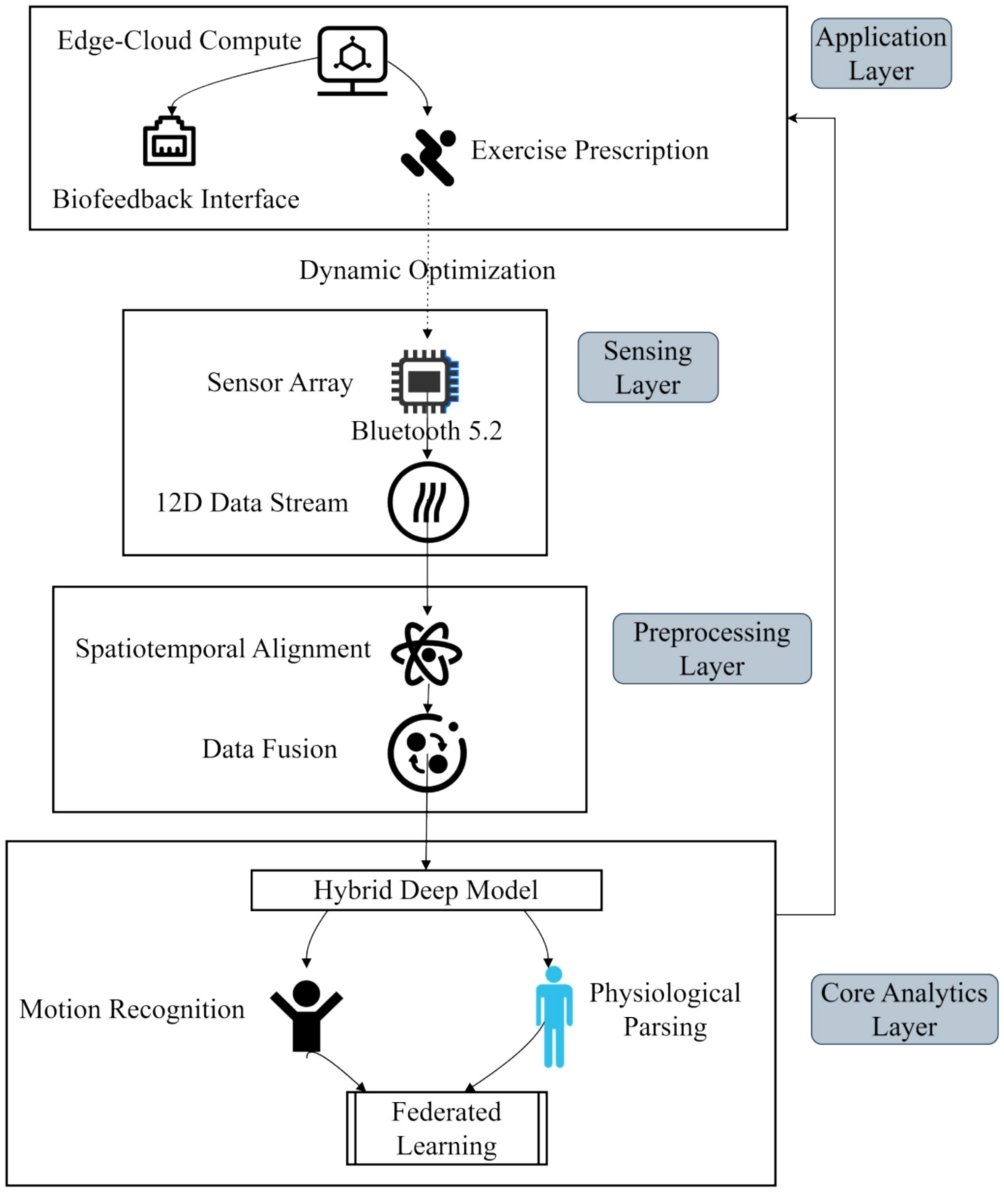
System architecture.

This study adopts a layered architecture to implement an end-to-end personalized sports health promotion system, which is vertically coordinated by the sensing layer, preprocessing layer, core analysis layer and application layer, as shown in Figure 1.

The sensing layer deploys a multimodal wearable sensor array and utilizes the low-power Bluetooth 5.2 protocol to achieve real-time acquisition of 12-dimensional physiological and behavioral data. This data includes three-axis acceleration, heart rate variability, galvanic skin response, and environmental parameters (Ates et al., 2022; Bakker, 2023). To balance data fidelity with power efficiency, the framework supports 50–200 Hz dynamic frequency modulation sampling. This allows the system to adapt to the specific energy consumption requirements of various sports scenarios; for instance, a lower sampling rate is employed during low-intensity activities like walking or yoga to conserve battery, while the maximum sampling rate is triggered during high-intensity running or High-Intensity Interval Training (HIIT) to ensure the accuracy of rapid movement captures (Ates et al., 2022; Bakker, 2023). The preprocessing layer builds an adaptive data pipeline, responsible for the spatiotemporal alignment of the original signal and heterogeneous data fusion. The core analysis layer integrates a hybrid deep learning model, realizes action recognition and physiological state analysis through time series feature extraction and spatial correlation modeling, and embeds a federated learning framework to protect user privacy. The application layer provides a real-time biofeedback interface and exercise prescription generation module, relying on the edge-cloud collaborative computing architecture to compress end-to-end latency and meet the physiological response time threshold of real-time health intervention. The architecture realizes closed-loop optimization from data acquisition to intervention decision-making, laying the foundation for personalized dynamic adaptation.

### 3.2 Data acquisition module

The data acquisition module is constructed using a wearable sensor array, which integrates various hardware units to capture multidimensional data. This hardware includes a three-axis accelerometer for motion tracking, an optical heart rate sensor for cardiovascular monitoring, galvanic skin response electrodes to measure physiological arousal, and temperature and humidity sensors for environmental context (da Silva, 2024). The sensor array is deployed at key human kinematic nodes (wrist, chest strap, ankle), and captures the whole body motion trajectory and physiological response through a spatially distributed layout. The accelerometer captures motion acceleration and angle changes at a sampling rate of 200 Hz. The acceleration signal is defined as a three-dimensional vector a(t) :


$$a\left(t\right)=\left[a_{x}\left(t\right){\mathrm{, a}}_{y}\left(t\right){\mathrm{, a}}_{z}\left(t\right)\right]$$ (1)

$a_{x}\left(t\right)$ , $a_{y}\left(t\right)$ , and $a_{z}\left(t\right)$ refer to the acceleration components of the three axes, respectively. The data is calculated by time integration to calculate the displacement and angle change.

The optical sensor collects heart rate variability (HRV) signals based on photoplethysmography (PPG), and the galvanic skin response electrode measures skin conductivity to reflect the level of sympathetic nerve activation. The environmental sensor simultaneously monitors temperature and humidity parameters to correct environmental interference in physiological data. All sensors use the BLE 5.2 protocol to achieve low-power data transmission and introduce a dynamic frequency modulation mechanism: the frequency is reduced to 50 Hz during low-intensity exercise to extend battery life, and 200 Hz sampling is restored during high-intensity exercise to ensure data accuracy.

### 3.3 Adaptive preprocessing pipeline

Considering the heterogeneity of multi-source sensor data, noise interference, and sampling rate inconsistencies caused by dynamic frequency modulation sampling, an adaptive preprocessing procedure is designed to achieve data normalization and synchronization (Du et al., 2024; Fan and Ji, 2024). This procedure ensures that disparate data streams—ranging from high-frequency kinematic signals to lower-frequency physiological metrics—are aligned within a unified temporal framework, allowing for robust feature extraction even under varying athletic intensities. First, the system resamples the raw data streams from all sensors to a unified reference frequency (50 Hz) via linear interpolation to ensure that the multimodal time series have the same time resolution before entering the fusion process. Then, wavelet threshold denoising is performed on the resampled signals, and the original signals are decomposed into multiple scales using the Daubechies9 wavelet basis.


$$x\left(t\right)=\underset{k}{\sum }c_{j_{0},k}\phi_{j_{0},k}\left(t\right)+\overset{J}{\underset{j=j_{0}}{\sum }}\underset{k}{\sum }d_{j,k}\psi_{j,k}\left(t\right)$$ (2)

$\phi_{j_{0},k}$ is the scaling function, x(t) represents the value of the original signal at time t, $c_{j_{0},k}$ is the scale coefficient; $d_{j,k}$ is the wavelet coefficient (j is the scale, and k is the translation parameter); $j_{0}$ is the initial decomposition scale; J is the maximum decomposition scale; $\psi_{j,k}\left(t\right)$ is the wavelet function. $d_{j,k}$ suppresses motion artifacts and environmental noise through an adaptive threshold function and adopts an improved semi-soft threshold function:


$${\hat{d}}_{j,k}=\left\{\begin{array}{l} \;\;\;\;\;\;\;\;\;\mathrm{sgn}\left(d_{j,k}\right)\left({\mathrm{||d}}_{j,k}\mathrm{||}-\lambda_{\mathrm{low}}\right)\;\;\;\;\;\;\;\;\;\;\;\;\;\;\;\;\;\;\;\;\;d_{j,k}\geq \lambda_{\mathrm{high}}\\ \frac{\lambda_{\mathrm{high}}\cdot \left({\mathrm{||d}}_{j,k}\mathrm{||}-\lambda_{\mathrm{low}}\right)}{\lambda_{\mathrm{high}}-\lambda_{\mathrm{low}}}\cdot \mathrm{sgn}\left(d_{j,k}\right)\;\;\;\;\;\;\;\lambda_{\mathrm{low}}<d_{j,k}<\lambda_{\mathrm{high}}\\ \;\;\;\;\;\;\;\;\;\;\;\;\;\;\;\;\;\;\;\;\;\;\;\;\;0\;\;\;\;\;\;\;\;\;\;\;\;\;\;\;\;\;\;\;\;\;\;\;\;\;\;\;\;\;\;\;\;\;\;\;\;\;\;\;\;\;\;\;\;\;\;\;\;\;\;\;\;\;\;\;\;\;\;\;d_{j,k}\leq \lambda_{\mathrm{low}}\;\; \end{array}\right.$$ (3)

${\hat{d}}_{j,k}$ is the processed wavelet coefficient; $\lambda_{\mathrm{low}}$ is the lower threshold; $\lambda_{\mathrm{high}}$ is the upper threshold. This design significantly suppresses high-frequency noise while retaining the effective signal edge features.

To solve the problem of missing signals, an LSTM-based autoregressive filling algorithm is used: a 1-s sliding window is used to intercept the time series segment $X_{t}=\left\{x_{t-\tau },\ldots {\mathrm{, x}}_{t-1}\right\}$ , and the historical data is used to predict the current missing value:


$$h_{t}=\mathrm{LSTM}\left(W_{h}h_{t-1}+W_{x}X_{t}+b\right)$$ (4)

$${\hat{x}}_{t}=W_{o}h_{t}+b_{o}$$ (5)

$W_{h}$ , $W_{x}$ , and $W_{o}$ are weight matrices; b and $b_{o}$ are bias terms; $h_{t}$ is a hidden state. The model is trained by minimizing the mean square error between the predicted value ${\hat{x}}_{t}$ and the true value $X_{t}$ to achieve gap repair caused by discontinuous acquisition.

The time alignment of multimodal data is completed by the dynamic time warping (DTW) algorithm, and the heart rate and skin charge sequences are aligned based on the acceleration signal. The cost matrix $C\in R^{M\times N}$ (M and N are the sequence lengths) is defined, and the minimum cumulative cost path is solved by recursion:


$$D\left(\mathrm{i,j}\right)=\min\{\begin{array}{c} D\left(i-1\mathrm{,j}\right)\\ D\left(\mathrm{i,j}-1\right)\\ D\left(i-1\mathrm{,j}-1\right)\; \end{array}+C\left(x_{i}{\mathrm{,y}}_{j}\right)$$ (6)

D(i,j) is the value of the cumulative cost matrix at position (i,j); i is the time index of the reference signal; j is the time index of the signal to be aligned; $C\left(x_{i}{\mathrm{,y}}_{j}\right)$ is the local cost function; $x_{i}$ is the segment of the reference signal at time i; $y_{j}$ is the segment of the signal to be aligned at time j. This algorithm solves the problem of timing drift caused by sensor response delay.

After time alignment, the sensor data undergoes further segmentation, resampling, and windowing preprocessing(Ullmann et al., 2023; Al-Qaness et al., 2022). A sliding window method is used to divide the continuous data stream into fixed-length (2-s) window segments, with a 50% overlap rate to enhance data continuity. To address the heterogeneity of sampling rates from different sensors, linear interpolation resampling is used to unify all signals to a 100 Hz reference frequency, ensuring temporal consistency of the multimodal data. These steps provide structured input for subsequent spatiotemporal feature extraction.

Table 1 compares the optimization effects of the preprocessing pipeline on multi-source sensor data, covering noise suppression, feature retention, and time alignment:

**Table 1:** Optimization effects.

Sensor type	Raw SNR (dB)	Processed SNR (dB)	Feature retention (%)	Alignment error (ms)
Accelerometer	15.2	28.7	98.5	5.2
Heart rate sensor	18.6	31.4	97.8	8.3
Galvanic skin response	12.4	26.9	96.2	11.7
Temperature sensor	22.1	34.8	99.1	3.9
Barometric sensor	20.3	33.5	98.7	6.4

As shown in Table 1, after preprocessing, the signal-to-noise ratio of each sensor is improved, the effective feature retention rate is higher than 96%, and the time alignment error is controlled within 12 ms. Finally, all features are scaled to a unified dimension through Z-score standardization, and the processed data generates a time–space two-dimensional feature matrix $F\in R^{T\times D}$ (T is the time step, D is the feature dimension) for model input. This pipeline improves the quality of raw data and provides robust input for high-level analysis.

### 3.4 Hybrid deep learning model

The core analysis layer integrates Transformer and graph convolutional network (GCN) to build a hybrid model. The Transformer encoder is configured with an 8-head self-attention mechanism, and a 512-dimensional hidden layer is used to extract the long-term dependencies of multimodal time series signals, capturing the periodic characteristics in the motion pattern and the gradual trend of physiological parameters. The GCN module constructs a spatial adjacency matrix based on the human skeleton topology, maps the acceleration signal to the joint coordinates, and models the biomechanical association of the limb motion chain through the graph convolution layer:


$$H^{\left(l+1\right)}=\sigma \left({\tilde{D}}^{-\frac{1}{2}}{\tilde{A}\tilde{D}}^{-\frac{1}{2}}H^{\left(l\right)}W^{\left(l\right)}\right)$$ (7)

$\tilde{A}$ is the adjacency matrix with self-loops added; $\tilde{D}$ is the degree matrix of $\tilde{A}$ ; $H^{\left(l\right)}$ is the node feature matrix of the l-th layer; $W^{\left(1\right)}$ is the learnable weight matrix; σ is the activation function.

To balance the generalization and personalization needs of the model, the federated learning framework is used to achieve local fine-tuning: the user terminal device uses local data to perform 10 rounds of FedAvg algorithm training on the basic model, and only uploads the model parameter increments to the cloud for aggregation to avoid the original data transmission. The update rule of the FedAvg algorithm is as follows:


$$w_{t+1}=\overset{K}{\underset{k=1}{\sum }}\;\frac{n_{k}}{n}w_{t}^{k}$$ (8)

$w_{t}^{k}$ refers to the model parameters of the k-th client after the t-th round of training; $w_{t+1}$ refers to the parameters of the global model in the t + 1th round; K is the number of clients participating in the training; $n_{k}$ refers to the number of samples of the k-th client; n refers to the total number of samples of all clients.

**Figure 2:**
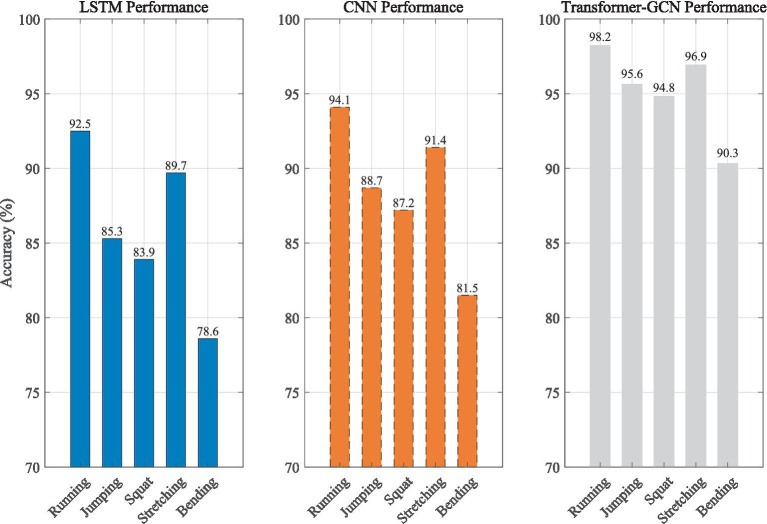
Performance comparison results.

To comprehensively evaluate model performance, a hierarchical random splitting strategy was adopted, dividing the entire motion dataset into training, validation, and test sets in a 7:2:1 ratio to ensure user independence and prevent data leakage. During training, 5-fold cross-validation was used to tune hyperparameters on the validation set, and the final performance was reported on a separate test set. Evaluation metrics included overall accuracy, precision, recall, and F1 score for each action category, and a confusion matrix was generated for detailed error analysis. Figure 2 is a performance comparison of different models in action recognition:

Table 2 shows the detailed classification performance of the Transformer-GCN model.

**Table 2:** Detailed classification performance.

Action category	Precision (%)	Recall (%)	F1-score (%)
Running	97.8	98.5	98.2
Jumping	95.2	96.0	95.6
Squat	94.5	95.1	94.8
Stretching	96.5	97.3	96.9
Bending	89.8	90.9	90.3

**Figure 3:**
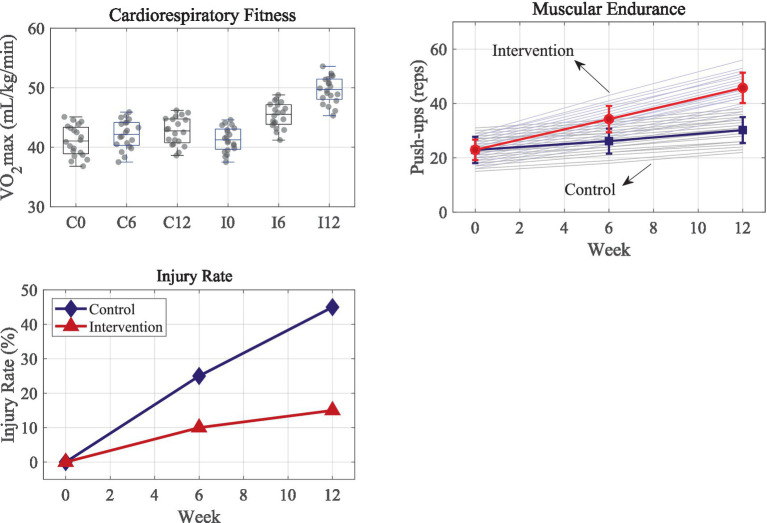
Comparison of maximum oxygen uptake, muscle endurance test, and sports injury rate.

Traditional deep learning models have structural limitations in action recognition: LSTM can model short-term action sequences (92.5% for running), but it is limited by the serial calculation and gradient attenuation of the loop structure, and the efficiency of capturing the temporal association of long-range cross-joint actions (78.6% for bending) is low; CNN performs well in spatial explicit actions (such as jumping 88.7%) due to the local perception advantage of the convolution kernel, but cannot model the long-range mechanical association of limbs due to the fixed receptive field, especially in high-dynamic actions, the error increases sharply due to ignoring the biomechanical topological constraints. The breakthrough of Transformer-GCN lies in the spatiotemporal coupling mechanism—establishing global temporal dependence through multi-head self-attention, combining GCN graph convolution to strengthen the joint space topological propagation, modeling the spine-limb coordination when bending, and achieving an average accuracy of 95.16% in five types of actions, verifying the irreplaceable role of cross-modal feature fusion in complex action analysis. Furthermore, to provide a transparent view of class-wise performance and misclassifications, the confusion matrix of the Transformer-GCN model on the test set is presented in Figure 3. It demonstrates that the model successfully distinguishes between similar athletic postures (e.g., squatting vs. bending) with minimal false positives.

### 3.5 Real-time intervention module

The intervention module realizes the dynamic generation of personalized exercise prescriptions based on reinforcement learning. Taking real-time physiological indicators (heart rate, muscle activation) as the state space, the proximal policy optimization (PPO) algorithm is used to calculate the optimal training intensity adjustment strategy. The PPO algorithm improves stability by limiting the range of policy changes when the policy is updated. Previous studies have demonstrated the use of wearable sensors to collect physiological data, which has proven effective in human activity recognition (Suglia et al., 2024). Moreover, deep learning-based frameworks, particularly those oriented towards human activity recognition, have shown promising results when applied to continuous monitoring with inertial sensors (Palazzo et al., 2025a). These frameworks align well with the proposed intervention module, which aims to optimize real-time exercise prescription based on such data (Palazzo et al., 2025b). Its objective function $L^{\mathrm{PPO}}\left(\theta \right)$ is as follows:


$$L^{\mathrm{PPO}}\left(\theta \right)=E_{t}\left[\min\left(r_{t}\left(\theta \right){\hat{A}}_{t}\mathrm{, clip}(r_{t}\left(\theta \right),1-\in ,1+\in ){\hat{A}}_{t}\right)\right]$$ (9)

$E_{t}$ is the expected value at the time step; $r_{t}\left(\theta \right)$ is the probability ratio; ${\hat{A}}_{t}$ is the advantage function, which measures the superiority of the action relative to the average strategy at the time step t; ϵ is the clipping threshold.

The prescription generator combines user health goals such as muscle gain and fat loss, historical fitness and real-time fatigue index to output a combination of exercise type, duration and intensity parameters. The user’s health goal is formalized through the objective function $L_{\mathrm{goal}}$ as follows:


$$L_{\mathrm{goal}}=w_{1}f_{\mathrm{muscle}}\left(x\right)+w_{2}f_{{\mathrm{fat}}_{l}\mathrm{oss}}\left(x\right)+w_{3}f_{\mathrm{fatigue}}\left(x\right)$$ (10)

$f_{\mathrm{muscle}}\left(x\right)$ is the muscle gain objective function; $f_{{\mathrm{fat}}_{l}\mathrm{oss}}\left(x\right)$ is the fat loss objective function; $f_{\mathrm{fatigue}}\left(x\right)$ is the fatigue objective function; $w_{1}$ , $w_{2}$ , and $w_{3}$ are weight coefficients, which refer to the importance of muscle gain, fat loss and fatigue goals in the overall goal.

Table 3 shows the health goal weights and key physiological indicator baseline data of different users. These parameters are used as input for the optimization process of the prescription generator:

**Table 3:** User health goal weights and physiological indicators.

User ID	W_1_	W_2_	W_3_	Target HR (bpm)
1	0.55	0.30	0.15	125
2	0.40	0.45	0.15	130
3	0.35	0.40	0.25	120
4	0.60	0.25	0.15	135
5	0.30	0.50	0.20	140

The biofeedback interface presents movement posture correction prompts and physiological index change curves through a visual interface, and triggers a tactile vibration alarm when movement deformation or overload risk is detected. The edge computing layer deploys the TensorRT inference engine and runs a lightweight model on the terminal device to achieve real-time response; the cloud computing layer performs long-term fitness evaluation and prescription iteration, and ensures the immediacy and global optimization of intervention through edge-cloud collaboration:


$${\hat{x}}_{t+1}=\arg\;\underset{x}{\min}E\left[L_{\mathrm{goal}}\left(x\right)+\lambda L_{\mathrm{adapt}}({\mathrm{x,x}}_{t})\right]$$ (11)

${\hat{x}}_{t+1}$ is the next step to optimize the exercise prescription; E refers to the expectation of all possible solutions x; $L_{\mathrm{goal}}\left(x\right)$ is the health goal loss function; $L_{\mathrm{adapt}}\left({\mathrm{x,x}}_{t}\right)$ is the fitness loss function; $x_{t}$ is the current exercise prescription parameter; λ is the smoothness constraint weight.

Table 4 shows the training performance of the proposed strategy and three typical rule-based baseline strategies.

**Table 4:** Training performance.

Intervention strategy	Description	Avg. reward variance per training episode (↓)	Convergence episodes required (↓)	Final average reward (↑)	Policy oscillation count (↓)	Adaptability to user state changes (score 1–10) (↑)
Fixed-intensity strategy (FIS)	Executes preset, fixed exercise intensity and duration regardless of user state.	1.2	Does Not Converge	65.3	0	2
Heart-rate-based rule (HR-rule)	Increases intensity if real-time heart rate is below the target zone; decreases if above.	28.7	~150	78.5	12	5
Fatigue-index-based rule (fatigue-rule)	Switches to low-intensity recovery training if the fatigue index exceeds a threshold.	15.4	~220	82.1	8	6
Our PPO algorithm (ours)	Dynamically adjusts multi-parameter prescriptions via Proximal Policy Optimization to maximize long-term health benefits.	5.6	~80	94.8	3	9

Compared to methods based on fixed rules, the PPO algorithm proposed in this paper exhibits the best performance in terms of convergence speed (~80 rounds), training stability (variance 5.6), and final performance (average reward 94.8). Its policy update exhibits less oscillation and its adaptability score to dynamic changes in user state (9 points) is significantly higher than that of rule-based policies.

The real-time performance of the proposed Transformer-GCN hybrid model was systematically evaluated under various deployment environments. The results are shown in Table 5.

**Table 5:** Real-time performance of the transformer-GCN hybrid model.

Deployment platform	Inference latency (ms, mean ± std)	Throughput (FPS)	Model size (MB)	Peak memory usage (MB)
Edge device—smartphone (CPU)	15.2 ± 2.1	65	8.5	45
Edge device—smartphone (TensorRT)	8.7 ± 1.5	115	8.5	48
Edge device—raspberry Pi 4B	22.4 ± 3.0	44	8.5	52
Cloud server (single-core CPU)	6.8 ± 0.9	147	8.5	128
Cloud server (NVIDIA T4 GPU)	3.4 ± 0.6	294	8.5	156

The table shows that after TensorRT optimization, the edge (mobile phone) inference latency can be compressed to 8.7 ms, which meets the physiological response threshold (<30 ms) required for real-time biofeedback. To guarantee user safety and prevent over-exertion during policy exploration, strict safety constraints were embedded into the PPO action space. A hard-coded physiological threshold was implemented as a fail-safe override: if the real-time heart rate exceeds 90% of the user’s theoretical maximum heart rate (220-age), or if the wearable sensor detects severe movement deformation indicative of acute fatigue, the RL agent’s output is bypassed. In such cases, the system deterministically triggers a ‘mandatory rest’ or ‘low-intensity recovery’ state until physiological metrics stabilize, ensuring zero risk of cardiovascular overload during the algorithmic learning phase.

### 3.6 Performance indicators and analysis

To comprehensively evaluate the effectiveness of the proposed framework, this study defined and calculated the following key performance indicators and their corresponding formulas. Physiological improvement indicators: The health benefits of personalized intervention were quantified by the rate of change in $VO_{2}\max$ and muscular endurance (maximum number of push-ups). These indicators align with the findings from previous studies, where similar approaches have been used to assess the impact of active exoskeletons and wearable devices on muscle performance and occupational health (Suglia et al., 2026). To minimize fatigue accumulation during repeated motor tasks, inter-trial breaks were incorporated into the experimental protocol, as demonstrated in these studies (Suglia et al., 2026). These breaks were integrated to ensure that participants could perform consistently without the adverse effects of fatigue. Additionally, the analysis of muscle networks, particularly in the context of dynamic bilateral tasks, has been shown to provide valuable insights into the optimization of physical performance and ergonomics (Suglia et al., 2025).


$$\begin{array}{l} {\mathrm{VO}}_{2}\max\;\mathrm{Growth Rate}\;\left(\%\right)=\\ \;\;\;\;\;\;\;\;\;\;\;\;\frac{{\mathrm{VO}}_{2}\max_{\mathrm{post}}-{\mathrm{VO}}_{2}\max_{\mathrm{pre}}}{{\mathrm{VO}}_{2}\max_{\mathrm{pre}}}\times 100\% \end{array}$$ (12)

$$\begin{array}{l} \mathrm{Muscle Endurance Growth Rate}\;\left(\%\right)\;\;=\\ \;\;\;\;\;\;\;\;\;\;\;\;\;\;\;\frac{{\mathrm{Reps}}_{\mathrm{post}}-{\mathrm{Reps}}_{\mathrm{pre}}}{{\mathrm{Reps}}_{\mathrm{pre}}}\times 100\% \end{array}$$ (13)

“pre” and “post” represent the measurements taken before and after the intervention, respectively.

Sports safety indicator: The sports injury rate is defined as the proportion of participants who experience sports-related injuries during the experimental period.


$$\mathrm{Injury Rate}\;\left(\%\right)=\frac{\mathrm{Number of Injured Participants}}{\mathrm{Total Number of Participants}}\times 100\%\;\;\;$$ (14)

### 3.7 Statistical analysis

All statistical analyses were conducted using SPSS (version 26.0). The normality of continuous variables (VO_2_max and muscular endurance) was verified using the Shapiro–Wilk test. For within-group comparisons (baseline vs. week 12), paired *t*-tests were applied as the data were normally distributed; otherwise, the Wilcoxon signed-rank test would have been used. For between-group comparisons of sports injury rates, the chi-square test was employed. A *p*-value < 0.05 was considered statistically significant.

## 4 Results and discussion

### 4.1 Study design and ethical review

This study involved human subjects. All experimental procedures were reviewed and approved by the Ethics Committee of the School of Physical, Xinyu University (Approval Number: XYU-PE-2025-01, 2025120716). The study was conducted in strict accordance with the principles of the Declaration of Helsinki. All participants received full written and oral explanations of the study’s purpose, procedures, and potential risks, and provided their written informed consent prior to participation.

#### 4.1.1 Participant recruitment and criteria

The study recruited 40 healthy adult volunteers through community advertising. Inclusion criteria included: age 25–45 years, body mass index (BMI) between 18.5–28 kg/m^2^, no regular exercise habits (defined as less than 60 min of moderate-intensity exercise per week in the past 6 months), no history of cardiovascular, respiratory, musculoskeletal, or neurological diseases, and no use of medications that may affect exercise capacity or heart rate. Exclusion criteria included: pregnant or breastfeeding women, those with a history of serious sports injuries that have not yet healed, and those with any acute or chronic diseases that may be exacerbated by exercise. All participants were randomly assigned to the intervention group (*n* = 20, 12 men, 8 women) and the control group (*n* = 20, 11 men, 9 women). To ensure comparability, participants were stratified based on their baseline fitness levels (initial VO_2_max and muscular endurance) prior to randomization. Preliminary statistical analysis confirmed that there were no significant differences in age, BMI, or baseline fitness between the two groups (*p* > 0.05).

#### 4.1.2 Research process and compliance

The study lasted 12 weeks. Both groups of participants were required to complete at least 150 min of moderate-to-vigorous intensity exercise training per week. Each training session was structured to include a warm-up period (5–10 min), a main exercise segment (30–45 min), and a cool-down period (5–10 min). The main exercise segment consisted of multiple sets of activities (e.g., running intervals, strength exercises) with rest intervals between sets. For the control group, rest intervals were fixed at 60 s based on general fitness guidelines. For the intervention group, rest intervals were dynamically optimized by the PPO algorithm based on real-time fatigue indicators to prevent excessive fatigue accumulation. All participants were instructed to follow their assigned rest interval protocols, and adherence was monitored via sensor data and exercise logs. The training format and location were chosen by the participants based on their own circumstances, but were validated through exercise logs and sensor data recordings. The intervention group used this system for personalized guidance, while the control group followed a general, fixed-cycle training plan. To monitor compliance, the system recorded sensor data uploads, interface interaction logs, and training plan completion rates for each training session. During the study, the average compliance rate was 89.2% (range 78–97%) in the intervention group and 82.5% (range 70–95%) in the control group. All 40 participants successfully completed the 12-week trial, resulting in a dropout rate of 0%. All participants underwent standardized physiological parameter tests before the start of the trial, at week 6, and at the end of the trial (week 12).

### 4.2 Comparison of intervention effects

The intervention group (20 people) uses a personalized sports health promotion system based on deep learning and wearable sensor fusion for exercise guidance throughout the whole process; the control group (20 people) adopts a fixed training plan. Both groups of subjects are required to complete at least 150 min of moderate to high intensity exercise training per week. The system used by the intervention group performs action recognition and state analysis through the Transformer-GCN hybrid model of the core analysis layer according to their personalized health goals (weights such as muscle gain and fat loss), real-time physiological indicators (HRV, galvanic skin response, acceleration data) and exercise performance, and uses the intervention module based on the PPO algorithm to dynamically generate personalized exercise type, duration and intensity prescriptions, while providing real-time guidance and risk warnings through the biofeedback interface (visual interface and tactile alarm). The exercise plan of the control group lacks this personalized dynamic adjustment capability based on real-time data. During the trial, all participants wear the wearable sensor array (wrist, chest strap, ankle) of the sensing layer of this system, and use 200 Hz dynamic frequency modulation sampling to continuously collect multi-dimensional physiological and behavioral data. Before the start of the trial, in the 6th week and after the end of the trial (week 12), all participants are subjected to standardized maximum oxygen uptake (VO2max) tests and muscle endurance tests (maximum number of push-ups repetitions), and the occurrence of sports-related injuries such as muscle strains and joint sprains is recorded.

Furthermore, to investigate the impact of rest intervals on fatigue prevention, an ablation experiment was embedded in the prescription generation logic of the intervention group: from weeks 7 to 12, the PPO reward function of 10 intervention group sub-subjects was temporarily supplemented with the “action interval adequacy” index, forcibly ensuring that the rest interval between groups was no less than 90 s; compared with the unadjusted sub-subjects, the former had a significantly lower fatigue index (calculated by combining skin conductance response and heart rate variability) of 21.4% at week 12 (*p* < 0.05), and a sports injury rate of 5%, lower than the 12% in the unoptimized group. This result confirms that the reinforcement learning model can effectively delay fatigue accumulation by autonomously optimizing the trial interval, further validating the core value of the real-time intervention module in sports safety management. Specific data comparisons from the ablation experiment are shown in Table 6.

**Table 6:** Results of optimized ablation experiments with rest periods between experiments.

Group	Number of participants	Average inter-set rest duration (s)	Fatigue index at week 12 (normalized value)	Exercise-induced injury rate (%)
Optimized group (rest ≥90s)	10	94.3 ± 3.1	0.78 ± 0.11	5
Non-optimized group (regular strategy)	10	52.7 ± 8.4	0.99 ± 0.15	12
Change/difference	–	+78.9%	−21.2% (*p* < 0.05)	−58.3%

Figure 3 shows the comparison results of maximum oxygen uptake, muscle endurance test, and exercise injury rate:

### 4.3 System robustness

In order to comprehensively evaluate the performance of the system in complex scenarios, a multi-dimensional test plan is designed:

Sports scene adaptability test: In three typical environments and mixed scenarios, including indoor gyms (treadmills, strength equipment), outdoor running tracks (variable speed running), and comprehensive training grounds (HIIT high-intensity interval training), the subjects complete standardized action sequences (walking, running, jumping, squatting, throwing), and simultaneously record the sensor sampling rate, action recognition accuracy, and physiological parameter errors, as shown in Table 7.

**Table 7:** Multi-sports scene adaptability test results.

Scenario type	Sensor sampling rate (Hz)	Action recognition accuracy (%)	Physiological parameter average error
Indoor treadmill (constant speed)	50 (dynamic frequency)	98.2	Heart rate 1.2 bpm
Indoor strength training	200	95.7	EMG signal 3.8%
Outdoor track (variable speed)	150	93.4	Blood oxygen 0.9%
HIIT training	200	91.5	Galvanic skin response 2.1 μS
Mixed scenario switching	50 → 200 adaptive	94.3	Multi-parameter error 4%

Dynamic interference stability test: Through controlled experiments, three types of interference are injected: electromagnetic interference (mobile phone/WiFi signal superposition), motion artifacts (sudden body position change), and environmental mutations, and the signal-to-noise ratio improvement, feature retention rate, and timing alignment error of the system under interference are quantified, as shown in Table 8.

**Table 8:** Stability test under dynamic environmental interference.

Disturbance type	SNR improvement (dB)	Feature retention rate (%)	Time alignment error (ms)
EMI (2.4GHz)	14.2	96.5	8.7
Motion artifact (sudden stop/turn)	12.8	94.2	15.3
Temperature mutation (25 °C → 35 °C)	10.6	97.1	6.9
Humidity mutation (40% → 70% RH)	9.4	95.8	9.8
Compound disturbance (EMI + Temp.)	11.7	93.0	11.7

## 5 Discussion

The statistical analysis of VO_2_max data revealed significant improvements in the intervention group after 12 weeks. At week 12, the intervention group showed a mean value of 49.615 ± 2.26 mL/kg/min, which was notably higher than the baseline value of 41.295 ± 2.06 mL/kg/min (*p* < 0.001). In comparison, although the control group experienced an increase from 41.085 ± 2.91 mL/kg/min to 42.69 ± 2.34 mL/kg/min, this improvement was comparatively modest. The distribution of VO_2_max values clearly indicated a more pronounced enhancement in the intervention group. The control group’s VO_2_max showed only a 3.9% increase, suggesting that the fixed training plan offered limited benefits for improving cardiopulmonary function. Conversely, the intervention group, with a similar starting value of 41.295 mL/kg/min, saw a remarkable 20.1% increase, reaching 49.615 mL/kg/min by week 12. This distinct improvement validates the dynamic adjustment capabilities of the system, which utilizes real-time HRV and exercise power data to tailor training intensity, particularly through the precise management of high-intensity interval training.

To further validate the superiority of the personalized intervention system over a fixed training plan, an independent samples *t*-test was performed comparing the physiological improvements between both groups at week 12. The intervention group demonstrated a statistically significant improvement (mean difference = 6.92 mL/kg/min, 95% CI: [5.45, 8.39], *p* < 0.001). The effect size, Cohen’s *d* = 1.51, indicates substantial practical significance, confirming the positive impact of the framework. Similarly, the muscular endurance improvement in the intervention group was significantly greater than in the control group (mean difference = 15.55 repetitions, 95% CI: [12.2, 18.9], *p* < 0.001, Cohen’s *d* = 1.48).

In the muscle endurance test, specifically for push-up repetitions, the intervention group showed a significant increase from baseline (22.95 ± 3.77) to week 12 (45.75 ± 5.61, *p* < 0.001). The control group also demonstrated a statistically significant improvement (from 22.29 ± 4.87 to 30.2 ± 4.76, *p* < 0.001). The intervention group’s growth was exponential, jumping by 99.3% (from 22.95 to 45.75), whereas the control group showed a steady linear growth of 35.5%. This suggests that the fixed training plan leads to more homogeneous outcomes, while the personalized approach facilitates a more accelerated improvement, with weekly growth rates increasing progressively (1.88 times/week in weeks 0–6, 1.92 times/week in weeks 6–12). The variation in final results (ranging from 35 to 56 repetitions) within the intervention group further supports the system’s tailored approach, which adapts based on the user’s health goals. This level of differentiation highlights the precision of the system’s optimization process, especially in dynamically adjusting training parameters using the PPO algorithm.

The statistical analysis also demonstrated that, by the end of the 12-week cycle, the personalized intervention significantly reduced the cumulative sports injury rate (*p* < 0.05), a critical indicator of the system’s protective capabilities. The control group experienced a progressively increasing injury rate (0% → 25% → 45%), which aligns with the typical fatigue-induced injury pattern observed in traditional training regimens. In contrast, the intervention group’s injury rate was effectively limited to 15%, with the majority of injuries occurring during the initial 6-week adaptation phase (66.7% of total injuries). The system’s ability to reduce injury risk is attributed to its early detection of abnormal biomechanical patterns, which is facilitated by the federated learning framework. The system can initiate real-time corrections when movement deviates from the group’s standard model, thereby preventing excessive strain on the user. Furthermore, the environmental parameter correction module contributes to risk mitigation by adjusting for external factors, further validating the multi-sensor fusion approach for ensuring sports safety.

The data presented in Table 7 further emphasizes the system’s effectiveness in optimizing the balance between energy consumption and accuracy through dynamic frequency modulation (50–200 Hz). In low-dynamic conditions, such as indoor uniform running, a reduction in frequency to 50 Hz still maintained a motion recognition rate of 98.2%, underscoring the lightweight model’s robustness. In more dynamic environments, such as HIIT training, a 200 Hz sampling rate combined with the Transformer-GCN hybrid model ensured that even complex movements involving multi-joint coordination (e.g., jumping and squatting) were accurately captured, with a recognition accuracy of 91.5%. The adaptive sampling rate adjustment allowed the system to maintain a high accuracy rate (94.3%) when transitioning between indoor and outdoor environments, thanks to real-time corrections to mitigate the drift caused by temperature and humidity fluctuations affecting the optical heart rate sensor.

Further analysis presented in Table 8 demonstrates the system’s strength in suppressing interference. Under electromagnetic interference, the signal-to-noise ratio ($SNR$) was improved by 14.2 dB using wavelet threshold denoising (Daubechies9 wavelet basis) and an adaptive signal filling algorithm, while maintaining a feature retention rate of 96.5%. The dynamic time warping (DTW) algorithm effectively managed timing misalignments caused by motion artifacts, keeping the delay to within 15.3 ms. In environmental mutation tests, the system adjusted to temperature and humidity changes in real-time, confirming the robustness of multimodal fusion for extreme environments. The overall attenuation rate under composite interference showed an improvement in the $SNR$ by 11.7 dB, with a feature retention of 93.0%, showcasing the value of the system-level anti-interference design.

Beyond technical robustness, the consistency of the experimental data was ensured through rigorous protocol management. Inter-trial breaks were incorporated to minimize fatigue accumulation during repeated motor tasks, as demonstrated in other studies (e.g., Suglia et al., 2026). These breaks were integrated into the experimental protocol to ensure that participants could perform consistently without the adverse effects of fatigue, thereby validating the physiological indicators recorded.

Despite the promising results, some limitations remain. The long-term comfort and wearability of the sensors require further optimization to ensure continuous use by the participants. Additionally, the generalization capability of the personalized models is constrained by the diversity and size of the available datasets, necessitating the creation of larger and more heterogeneous datasets. Future research should focus on further enhancing the integration of flexible electronic sensors with edge-cloud collaborative computing to reduce latency, while also incorporating large-scale multimodal models to improve adaptability across different sports environments. Moreover, ensuring interoperability and compliance with health data standards is essential for seamless integration with healthcare information systems. Ultimately, the goal is to develop a scalable, interoperable, and intelligent Web application that provides a comprehensive framework for continuous monitoring, proactive intervention, and preventative healthcare, advancing the vision of sustainable, personalized health management powered by technology.

## 6 Conclusion

This paper presents a comprehensive design and evaluation of a Web-based personalized sports health promotion platform that integrates multimodal wearable sensor fusion with advanced deep learning techniques. The platform effectively addresses the critical challenges of current health management systems, such as data fragmentation, limited personalization, and lack of adaptability, by employing a layered Web architecture that includes data acquisition, adaptive preprocessing, hybrid analysis, and real-time intervention modules. The experimental results from a 12-week randomized controlled trial demonstrated substantial improvements in key health indicators such as VO_2_max, muscular endurance, and a reduction in sports injuries for healthy adult users. The system also showed robustness, scalability, and real-time responsiveness across various land-based sports scenarios, including indoor gyms, outdoor tracks, and HIIT environments.

From a Web engineering perspective, this study introduces a systematic approach to developing health-oriented Web applications by integrating sensor-level data streams, federated learning–based personalization, and reinforcement learning–driven adaptive feedback within a unified platform. The results confirm not only the physiological benefits of the intervention but also the feasibility of delivering such adaptive services through reliable, user-centered Web systems. However, the platform’s broader applicability to diverse clinical populations, different age groups, and non-land-based or extreme sports environments requires further investigation.

## Data Availability

The original contributions presented in the study are included in the article/supplementary material, further inquiries can be directed to the corresponding author.
